# Essential Nucleoid Associated Protein mIHF (Rv1388) Controls Virulence and Housekeeping Genes in *Mycobacterium tuberculosis*

**DOI:** 10.1038/s41598-018-32340-2

**Published:** 2018-09-21

**Authors:** Nina T. Odermatt, Claudia Sala, Andrej Benjak, Stewart T. Cole

**Affiliations:** École Polytechnique Fédérale de Lausanne, Global Health Institute, Station 19, 1015 Lausanne, Switzerland

## Abstract

Tight control of gene expression is crucial for *Mycobacterium tuberculosis* to adapt to the changing environments encountered when infecting or exiting human cells. While three nucleoid associated proteins (NAPs) EspR, HupB and Lsr2 have been investigated, the role of a fourth, the mycobacterial integration host factor (mIHF), remains elusive. Here, we report a multidisciplinary functional analysis that exploits a conditional mIHF mutant. Gene silencing was bactericidal and resulted in elongated cells devoid of septa, with only one nucleoid. ChIP-sequencing identified 153 broad peaks distributed around the chromosome, which were often situated upstream of transcriptional start sites where EspR also bound. RNA-sequencing showed expression of 209 genes to be heavily affected upon mIHF depletion, including those for many tRNAs, DNA synthesis and virulence pathways. Consistent with NAP function, mIHF acts as a global regulator by directly and indirectly controlling genes required for pathogenesis and for housekeeping functions.

## Introduction

Bacterial gene expression is tightly controlled and influenced by environmental cues. In the case of the human pathogen *Mycobacterium tuberculosis*, which has to adapt to a hostile milieu upon phagocytosis by a macrophage, these stimuli include acidic pH, reactive oxygen species, nutrient limitation, fatty acid availability or the presence of lytic enzymes^1^. It is therefore crucial to comprehend gene expression to understand bacterial growth and survival. The regulons of traditional transcription factors comprise from one to tens of target genes. Nucleoid associated proteins (NAPs) on the other hand act at the global level impacting expression of hundreds of genes often by shaping chromatin architecture^2^. In *Escherichia coli*, more than twelve NAPs have been characterized so far. Their cellular abundance fluctuates during the different growth phases^3^ and each NAP targets a specific set of genes^4^. In contrast, only four NAPs have been reported to date in *M. tuberculosis*. HupB is essential for growth in macrophages and for iron acquisition^5^ and Lsr2 is an *E. coli* H-NS-like protein that preferentially binds to AT-rich sequences^6^. EspR regulates secretion of the main virulence factors of *M. tuberculosis*^7^ by controlling expression of the ESX-1 related *espACD* operon among others^8^. These three NAPs all have multiple binding sites on the *M. tuberculosis* chromosome and regulate the vast majority of genes. The overall picture of the regulatory network is unclear, and the role of the fourth NAP, mycobacterial integration host factor mIHF, has not been defined yet.

Discovered as being essential for mycobacterial phage L5 integration into the *M. smegmatis* genome^9^, mIHF was therefore named after the *E. coli* counterpart, despite the two genes and their respective proteins showing no sequence similarity. mIHF is highly conserved among the *Mycobacterium* genus and even *M. leprae*, with its reduced genome, possesses a copy of *mihF*^10^, and orthologs occur in many other Actinobacteria. The *mihF* (*rv1388*) gene in *M. tuberculosis* was initially predicted to be 573 bp-long and to encode a ~20 kDa protein^11^. More recently, based on comparative genomics, Mishra and colleagues proposed that mIHF of *M. tuberculosis* contains only 105 amino acid residues^12^ and this is supported by proteomics analysis with mIHF appearing among the top ten most abundant proteins of *M. tuberculosis*^13–15^. It has been reported that mIHF binds to linear and supercoiled DNA and enhances topoisomerase activity^12^.

The *mihF* gene was predicted to be essential for *in vitro* growth with glycerol or cholesterol as carbon sources by *Himar*-1 based transposon mutagenesis^16^, thus suggesting an important regulatory role in *M. tuberculosis* metabolism. In this study, we investigated the biological function of mIHF thoroughly with the help of a conditional knockdown (cKD) mutant. We show that mIHF is indeed essential for growth and affects protein and nucleic acid synthesis. Cells depleted of mIHF displayed aberrant morphology, and nucleoid segregation, before dying. The effect of mIHF depletion was investigated using ChIP-seq and RNA-sequencing thereby demonstrating that this NAP impacts *M. tuberculosis* gene regulation pleiotropically by controlling expression of housekeeping as well as virulence genes.

## Results

### mIHF is an abundant cytosolic protein

**Figure 1:**
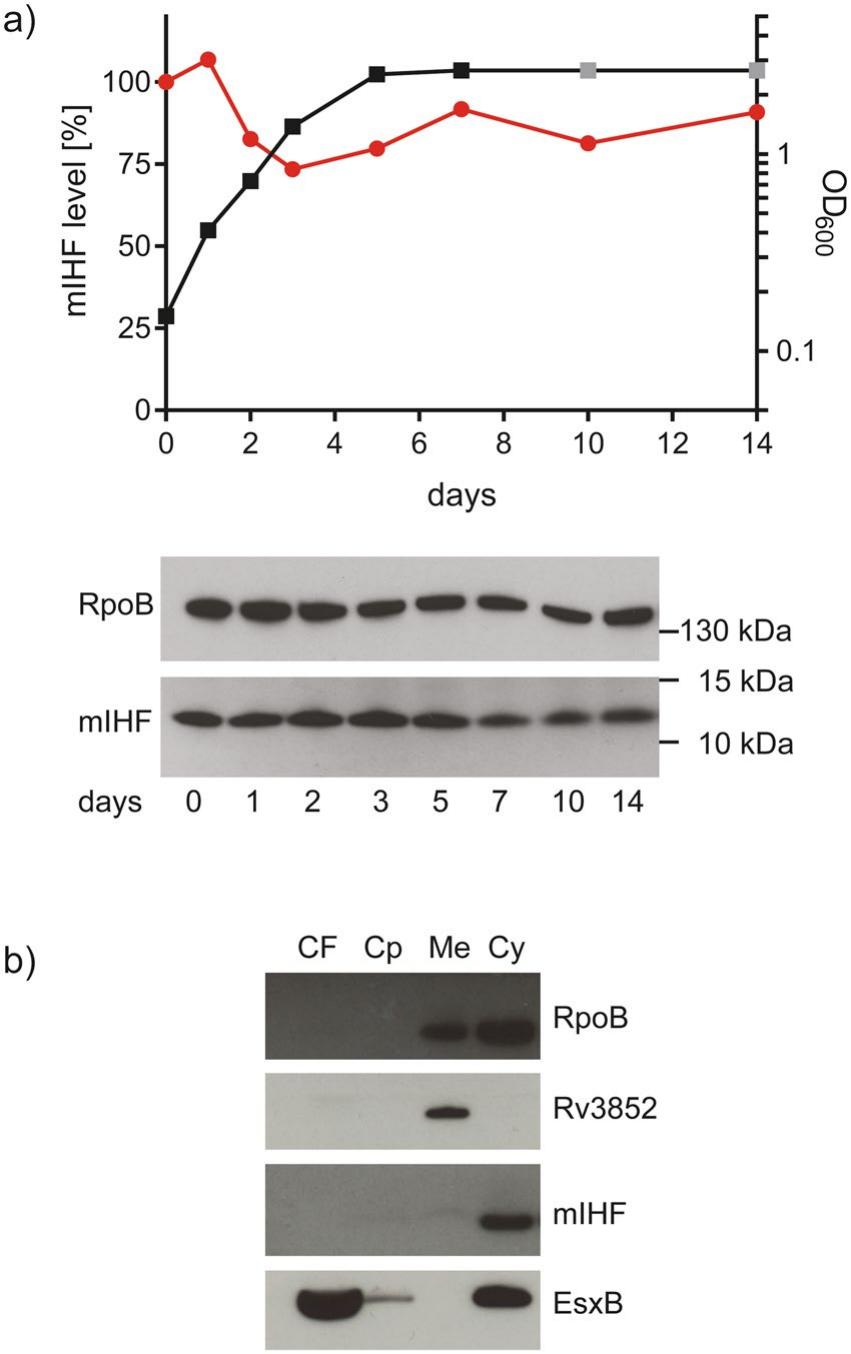
Expression and localization of mIHF. (**a**) Time-course analysis by immunoblot of mIHF levels (red bullets, left y-axis), and optical density at 600 nm (OD_600_, black squares, right y-axis) of H37Rv from exponential to stationary phase. Protein levels were calculated by density analysis of the image below, relative to RpoB and to the first time point (day 0). OD_600_ at days 10 and 14 (grey square) were set to the same value as day 7, as the culture formed aggregates typical of *M. tuberculosis* in stationary phase, which prohibited proper measurement of OD_600_. b) Immunoblot of culture filtrate (CF), capsular (Cp), membrane (Me) and cytosolic (Cy) fractions of H37Rv. Antibodies used are indicated to the right.

While some NAPs are present throughout the entire growth cycle of a bacterium, others peak at certain stages. The mIHF protein was identified at approximately 12 kDa by immunoblotting and a time-course analysis of protein levels showed that it is constantly present from the exponential to stationary phase with little fluctuations (Fig. 1a). To probe where mIHF is localized inside the bacterial cell, *M. tuberculosis* H37Rv cell extracts were fractionated prior to subsequent immunoblotting. Proteins RpoB, Rv3852^17^ and EsxB were used as positive controls for the cytosol, membrane and secreted fractions, respectively. The mIHF protein was detected in the cytosol only (Fig. 1b).

### Transcription start site identification and conditional knockdown mutant generation

To explore the regulatory function of mIHF, we constructed an *mihF* conditional knockout mutant after first localizing the transcription site(s) since it had been suggested that *mIHF* is shorter than originally annotated^12^. Transcripts were analysed by rapid amplification of cDNA ends (5′-RACE) and three potential transcription start sites (TSS) were detected (Fig. S1). The third of these, TSS3, situated 167 bp downstream of the currently annotated translation start site, is preceded by a TANNT -10 motif, shared by most promoters of *M. tuberculosis*^18^. From multiple mIHF sequence alignments and the presence of an appropriately positioned ribosome binding site (GGAGGAA)^19^, residue 86 of the original annotation was inferred to be the initiation codon (Fig. S1), as recently proposed^12^, and this will be referred to as *mihF*-86. An in-depth discussion about the *mihF* initiation codon is available in supplementary text 1.

Next, we employed gene replacement to remove the annotated, full-length *mihF* gene from the chromosome. After confirming the nature of the merodiploid strain, the second crossing over event, which led to the in-frame deletion of the gene, was only successful when a copy of *mihF* was provided *in trans* under the control of the repressible TET-PIP OFF system^20^. Furthermore, it was necessary to include the two downstream genes, *gmk* and *rpoZ*, on the complementing construct in order to delete the chromosomal *mihF* gene and therefore obtain the conditional *mihF*-cKD mutant. The mutation was confirmed by Southern blotting (Fig. S2).

Addition of anhydrotetracycline (ATc) to the *mihF*-cKD mutant complemented with the original full-length *mihF* caused no variation in mIHF protein abundance, nor in growth dynamics (data not shown). However, controllable complementation was obtained on introduction of plasmid pNO63, carrying *mihF*-86, thus confirming that this represents the *bona fide mihF* gene (named *mihF* hereafter).

### *mihF* is essential for growth and survival of *M. tuberculosis*

**Figure 2:**
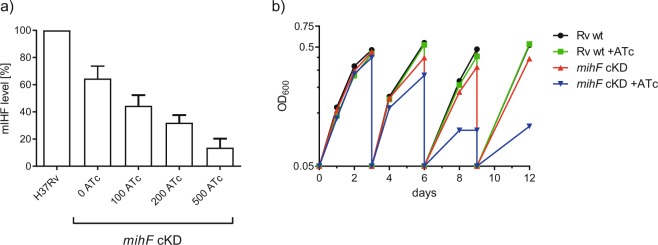
*mihF* silencing by ATc. (**a**) mIHF levels detected by immunoblot normalized to RpoB and relative to H37Rv wildtype. ATc concentration is indicated in ng ml^−1^. (**b**) Growth curves of *mihF*-cKD and H37Rv wild type strains with 600 ng ml^−1^ ATc and without ATc. Cultures were diluted to OD_600_ = 0.05 every 3 days.

Addition of increasing concentrations of ATc to cultures of the *mihF*-cKD mutant allowed titration of mIHF levels (Fig. 2a, S3). In the absence of ATc, the conditional mutant produced lower levels of mIHF than the wild type strain (approximately 30% less), indicating that the controllable *ptr* promoter is weaker than the natural one. The level of mIHF was reduced to less than 10% upon addition of 500 ng ml^−1^ ATc. Of note, it was necessary to dilute the *mihF*-cKD cultures at least twice to see a reduction of the growth rate of the mutant compared to the parental strain or to the uninduced *mihF*-cKD strain (Fig. 2b). As expected, ATc had no impact on the growth of the parental H37Rv strain.

**Figure 3:**
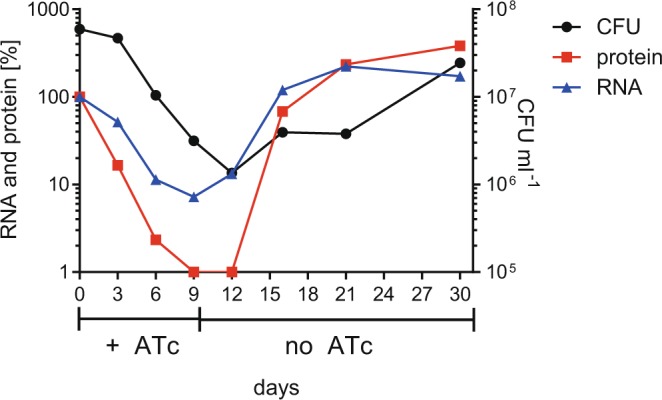
Effect of *mihF* silencing. *mihF* mRNA, mIHF protein levels and colony forming units in the presence of ATc (day 0–9, diluted every three days) and under permissive conditions (from day 9).

Figure 3 shows the viability of the *mihF*-cKD strain upon repression of *mihF* expression for 9 days, followed by three weeks under permissive conditions. While *mihF* RNA and mIHF protein levels rapidly decreased during the first three days, the number of colony forming units (CFU) dropped at day 6, proving that depletion of mIHF had a bactericidal effect. When ATc was removed from the bacterial culture, RNA production resumed, whereas a three-day lag was noticed before mIHF protein could be detected. Similarly, viable counts increased after a three-day lag under permissive conditions.

### Impact of mIHF depletion on macromolecular synthesis

**Figure 4:**
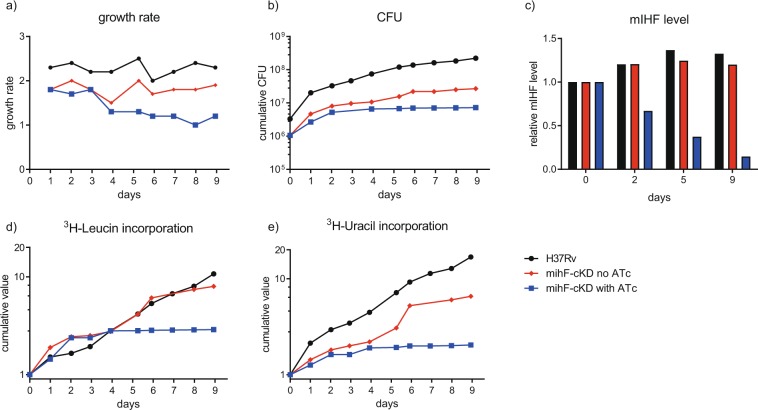
Tritium labelled-leucine and -uracil incorporation. (**a**) Growth rate of H37Rv wild type and *mihF*-cKD with and without ATc. (**b**) Cumulative CFU ml^−1^ of the three strains. (**c**) mIHF levels at days 0, 2, 5 and 9 measured by immunoblot, relative to day 0 for each strain. (**d**) Leucine and uracil (**e**) incorporation relative to day 0.

To examine if *de novo* protein and nucleic acid (RNA plus DNA) production was affected by mIHF-depletion, the incorporation of ^3^H-labelled leucine and uracil into nucleic acids and proteins, respectively, by the *mihF*-cKD strain was monitored over nine days in non-permissive conditions. Figure 4a shows that the growth rate of the mutant strain without ATc was lower than that of H37Rv, and then further decreased in the presence of ATc. Likewise, the total number of CFU increased from 5*10^6^ to 2*10^8^ CFU ml^−1^ for H37Rv after nine days, while *mihF*-cKD without ATc started at 1.05*10^6^ and later attained 2.68*10^7^ CFU ml^−1^ (Fig. 4b). In contrast, growth of the *mihF*-cKD mutant in the presence of ATc reached a plateau after four days and only increased to 7*10^6^ CFU ml^−1^ at day 9. While the mIHF protein levels rose slightly in the H37Rv and *mihF*-cKD strains without ATc, the *mihF*-cKD mutant already showed lower mIHF protein levels at day 2 after silencing and mIHF abundance dropped to ~40% at day five (Fig. 4c), when growth was arrested and nucleic acid as well as protein synthesis came to a halt (Fig. 4d and e). Depletion of mIHF had therefore a pleiotropic effect on *M. tuberculosis* physiology, as it severely compromised DNA, RNA and protein synthesis.

### mIHF-depleted bacteria are elongated and do not form septa

**Figure 5:**
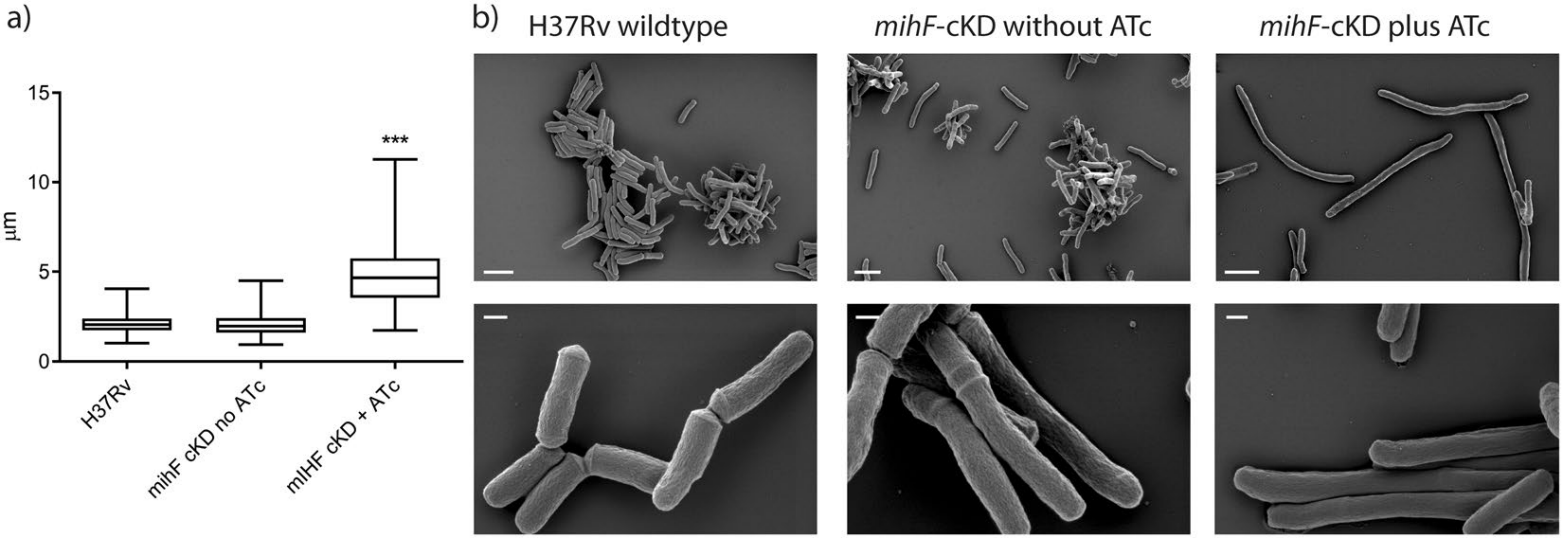
Cell morphology of mIHF-depleted bacteria. (**a**) Cell length measured by surface electron microscopy. *mihF*-cKD cells grown with ATc for 9 days are twice as long as H37Rv wild type and *mihF*-cKD grown in the absence of ATc (***p < 0.0001. Whiskers represent minimum and maximum values, median is plotted at the middle of the box). (**b**) Scanning electron micrographs of H37Rv parental strain and *mihF*-cKD strains with and without ATc. Bar represents 2 µm on the upper panel and 200 nm on the lower panel.

Scanning electron microscopy allowed investigation of the ultrastructure of *mihF*-cKD mutant cells. Cells depleted of mIHF were more than twice as long as the H37Rv parental strain and the *mihF*-cKD mutant grown without ATc (Fig. 5a). The average lengths for the wild type and *mihF*-cKD strain without ATc were 2.13 ± 0.53 µm and 2.09 ± 0.67 µm, respectively, whilst *mihF*-cKD cells with ATc were 4.98 ± 1.96 µm long. In addition, most of the induced *mihF*-cKD cells longer than 3 µm showed no ridge formation, which is indicative of the lack of an underlying septum^21^. Conversely, ridges were clearly visible in the wild type and in *mihF*-cKD cells without ATc (compare the lower panel of H37Rv and *mihF*-cKD plus ATc in Fig. 5b). Apart from the elongated, septum-less phenotype, no other abnormal shape was observed (i.e. no branching, swelling or bending) after depletion of mIHF.

**Figure 6:**
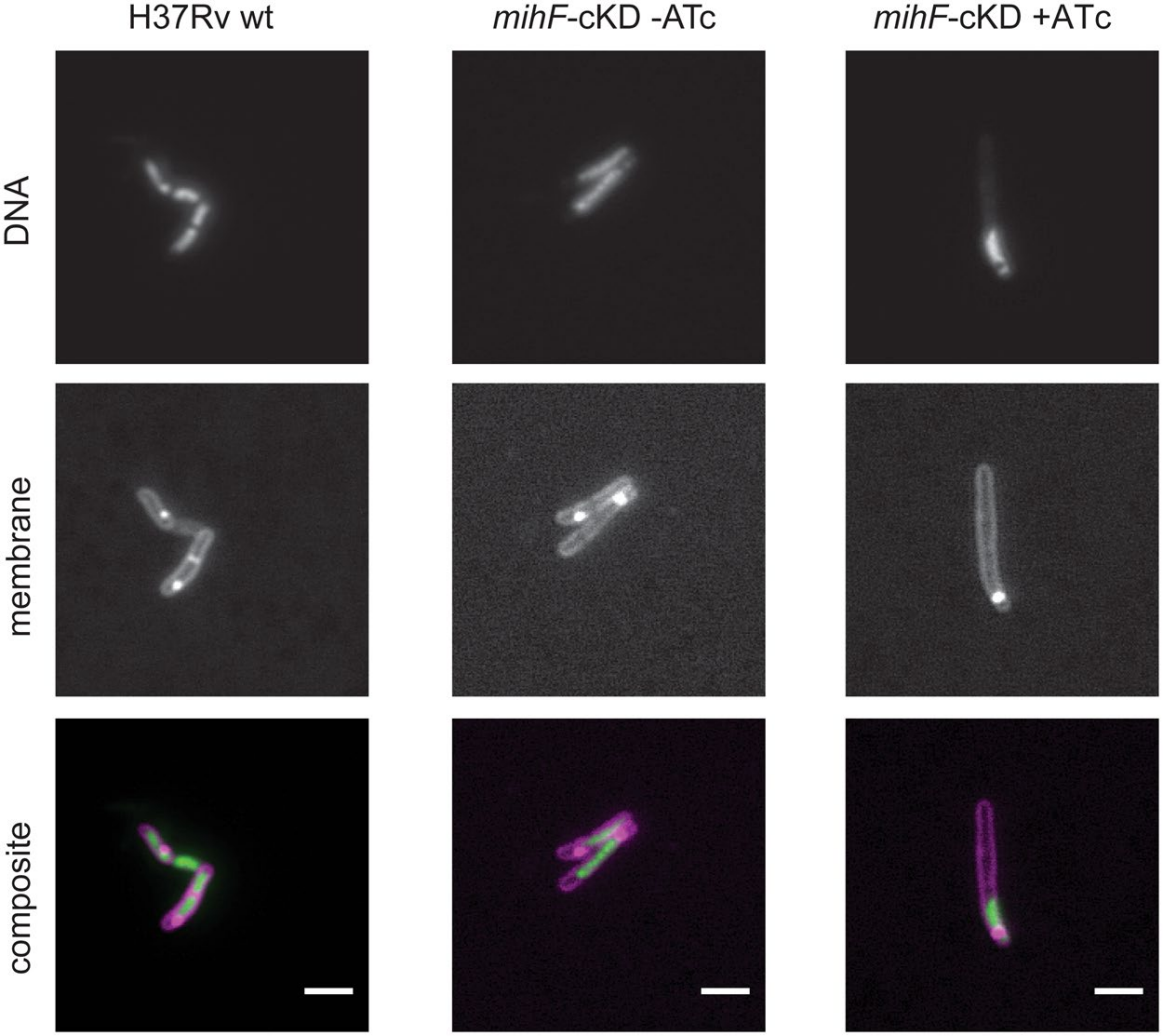
Septum and nucleoid positions. Fluorescence microscopy images of H37Rv wild type, *mihF*-cKD with and without ATc. DNA was stained with SYTO9, membranes with FM4–64. Scale bars represent 2 µm.

Fluorescence microscopy confirmed that most of the long mIHF-depleted cells did not generate septa and additionally showed that they contained only one nucleoid (Fig. 6). On the other hand, in septum-containing cells (wild type and *mihF*-cKD grown without ATc), DNA was present in each compartment (Fig. 6). Thus, mIHF is necessary for septum formation and normal cell division as well as for DNA segregation in *M. tuberculosis*.

### mIHF has a broad impact on the *M. tuberculosis* transcriptome

**Figure 7:**
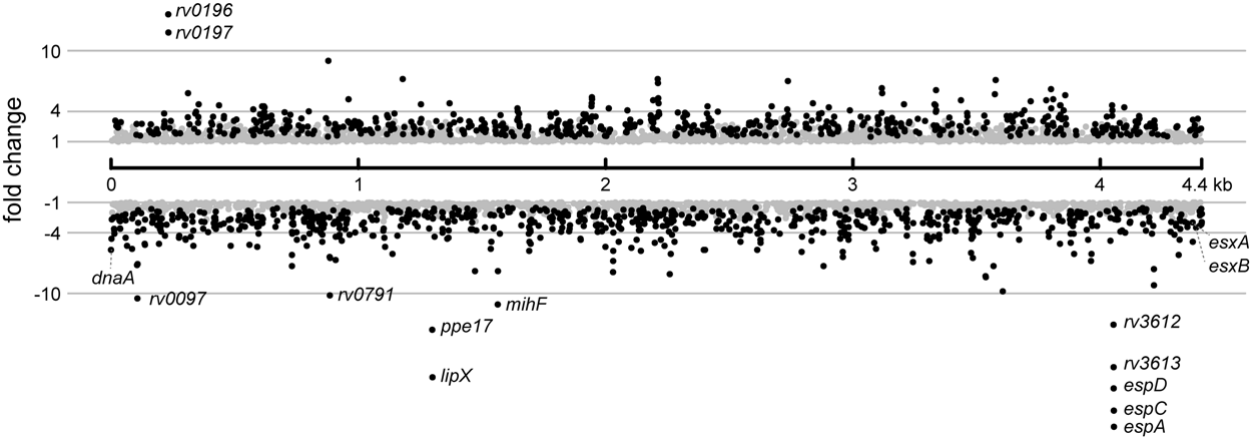
mIHF controlled genes. (**a**) Differentially expressed genes identified in mIHF-depleted relative to non-depleted *M. tuberculosis* cultures are plotted at their genomic position. Grey dots represent genes with a false discovery rate (FDR) higher than 1%, black dots are significantly deregulated genes with FDR <1%. Interesting genes discussed further in the text are labelled.

**Figure 8:**
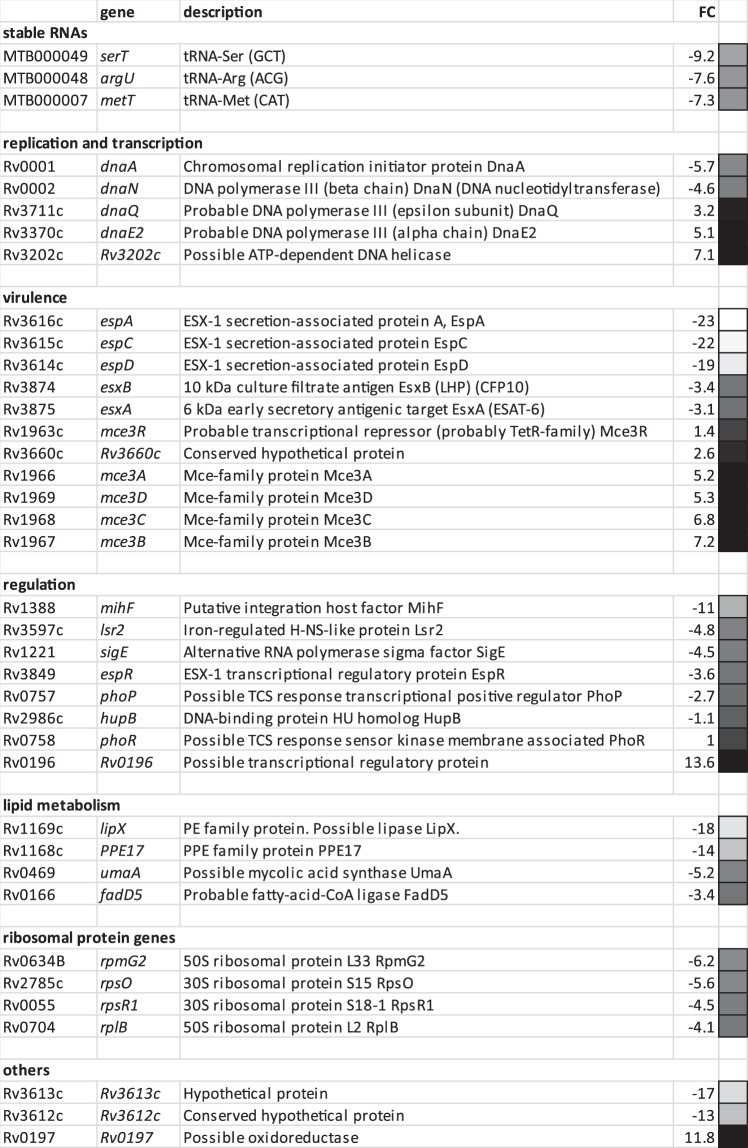
Selected top-scoring and differentially expressed genes in *mihF*-cKD upon ATc treatment. FDR <1%, fold change = FC, light squares represent downregulated and dark squares upregulated genes.

To characterize the mIHF-dependent regulon, global gene expression analysis was performed by RNA-seq and the data subjected to gene ontology (GO) analysis to identify biological processes. Effects of the inducer on the transcriptome were ruled out by comparing the transcription profile of strain H37Rv in the presence and absence of ATc. Using a false discovery rate (FDR) below 1% and a change cut-off >2-fold, no differentially expressed genes were found (Supplementary Dataset 1). Likewise, integration of the complementing plasmid did not affect gene expression significantly (Supplementary Dataset 1). The transcriptome of the *mihF*-cKD mutant strain in the presence and absence of ATc was then analysed using the same cut-off values. Depletion of mIHF had a broad impact on the global transcriptome as 679 downregulated and 464 upregulated genes were detected (Supplementary Dataset 1). By increasing the threshold to >4, the number of deregulated genes was limited to 150 downregulated and 59 upregulated (Supplementary Dataset 1). Figure 7 displays the distribution of the deregulated genes throughout the *M. tuberculosis* genome: no clustering was observed. The most severely affected genes are listed in Fig. 8 and discussed in greater detail below.

Strikingly, the operon showing the maximum repression was *espACD*, which is required for the ESX-1 type VII secretion system to function in *M. tuberculosis*. Additionally, various other genes encoding components of the Type VII secretion systems ESX-1, ESX-2 and ESX-5, and their substrates (EsxA, EsxB, EsxC, EsxD, EsxM, EsxN), were expressed at a lower level. mIHF has therefore a major impact on expression of virulence-related secretion systems. The second most downregulated operon was *rv1168*-*rv1169*, coding for PPE17 and LipX, respectively.

The majority of the stable RNAs, notably 23 out of 45 tRNAs and several small regulatory RNAs, were repressed by more than 2-fold, as well as some ribosomal protein genes (Fig. 8). Expression of the genes for the replication initiator protein DnaA and for the beta chain of the DNA polymerase III, DnaN, were >4-fold downregulated (Fig. 8). These results indicate that mIHF is involved in controlling transcription of housekeeping genes needed for protein and DNA synthesis.

Conversely, the most upregulated operon was *rv0196-rv0197*, coding for a transcriptional regulatory protein and a possible oxidoreductase, followed by two conserved hypothetical proteins (*rv0784*, *rv1057*) and the virulence-associated *mce3* operon. Moreover, several genes encoding transcription factors were found to be deregulated. For instance, expression of *lsr2*, *sigE* and *espR*, which may act downstream of mIHF, thus amplifying the regulatory signal, was diminished. The *mihF* gene itself was more than 11-fold repressed, confirming the silencing effect of ATc.

GO analysis revealed the most downregulated genes (p < 0.05) to be associated with host-pathogen interaction, fatty acid metabolism, pathogenesis, ribosomes, transcription factors, DNA binding proteins and DNA replication initiation (Supplementary Dataset 1). The GO categories related to upregulated genes were associated with DNA damage such as DNA duplex unwinding, DNA repair and exonuclease activity.

### mIHF is a nucleoid associated protein in *M. tuberculosis*

**Figure 9:**
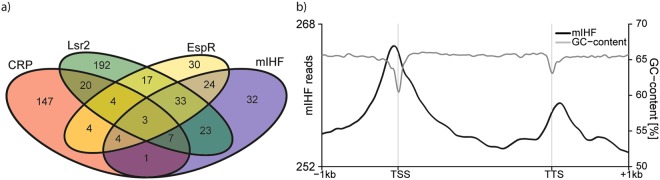
Binding sites of global transcription factors in *M. tuberculosis*. (**a**) Overlap of binding sites between CRP, Lsr2, EspR and mIHF. (**b**) mIHF binding peaks relative to the transcriptional start site (TSS) and transcriptional termination site (TTS), y-axis indicates number of reads. Black profile depicts mIHF binding in exponential phase (“mIHF”), grey profile shows GC content of the H37Rv genome (“GC-content”, right y-axis).

To evaluate if mIHF directly regulates the differentially expressed genes found in RNA-seq, mIHF binding to the *M. tuberculosis* chromosome was investigated by chromatin immunoprecipitation followed by high-throughput sequencing (ChIP-seq). Exponentially growing H37Rv wild type cells as well as mIHF-depleted *mihF*-cKD mutant cells were assessed. Upon analysing the ChIP-seq results from exponentially growing wild type cells (Supplementary Dataset 1), we noticed that many mIHF peaks overlapped the previously published EspR binding sites^7^. For the sake of consistency, EspR ChIP-seq was then repeated on the same H37Rv sample used for mIHF ChIP-seq. 162 EspR binding sites were detected, of which 128 had been previously described^7^ and the others were found close by (Fig. S4a for Pearson’s correlation coefficient, PCC). mIHF bound to 153 loci in exponential phase (Supplementary Dataset 1), whereas 124 sites were contacted upon depletion of the protein (Supplementary Dataset 1). 62 mIHF binding sites were occupied in exponentially growing cells and upon mIHF depletion (PCC of 0.67, Fig. S4), and 64 of the 153 loci were shared with EspR as well. The overlap between EspR and mIHF peaks was higher in exponential phase cells (PCC of 0.6) than after mIHF depletion (PCC of 0.44, Fig. S3). We further integrated the profiles of CRP (cAMP receptor protein) and Lsr2, with 191 and 305 binding sites, respectively. While a marked overlap was observed between the regions bound by the three NAPs (Lsr2, EspR and mIHF), CRP seemed to bind at different loci (Fig. 9a).

The GC content of regions where EspR and/or mIHF bound ranged between 58% and 60%, which is significantly lower than the genome average of ~66%, (p < 0.0001). This is consistent with the preferential location of mIHF binding near the transcriptional start sites (TSS, Fig. 9b). Indeed, mIHF-bound loci were mostly situated immediately upstream of the TSS, and a minor fraction was found after the transcriptional termination site (Fig. 9b). Moreover, mIHF binding sites appeared as broad rather than as sharp peaks in exponentially growing as well as in mIHF-depleted cells.

**Figure 10:**
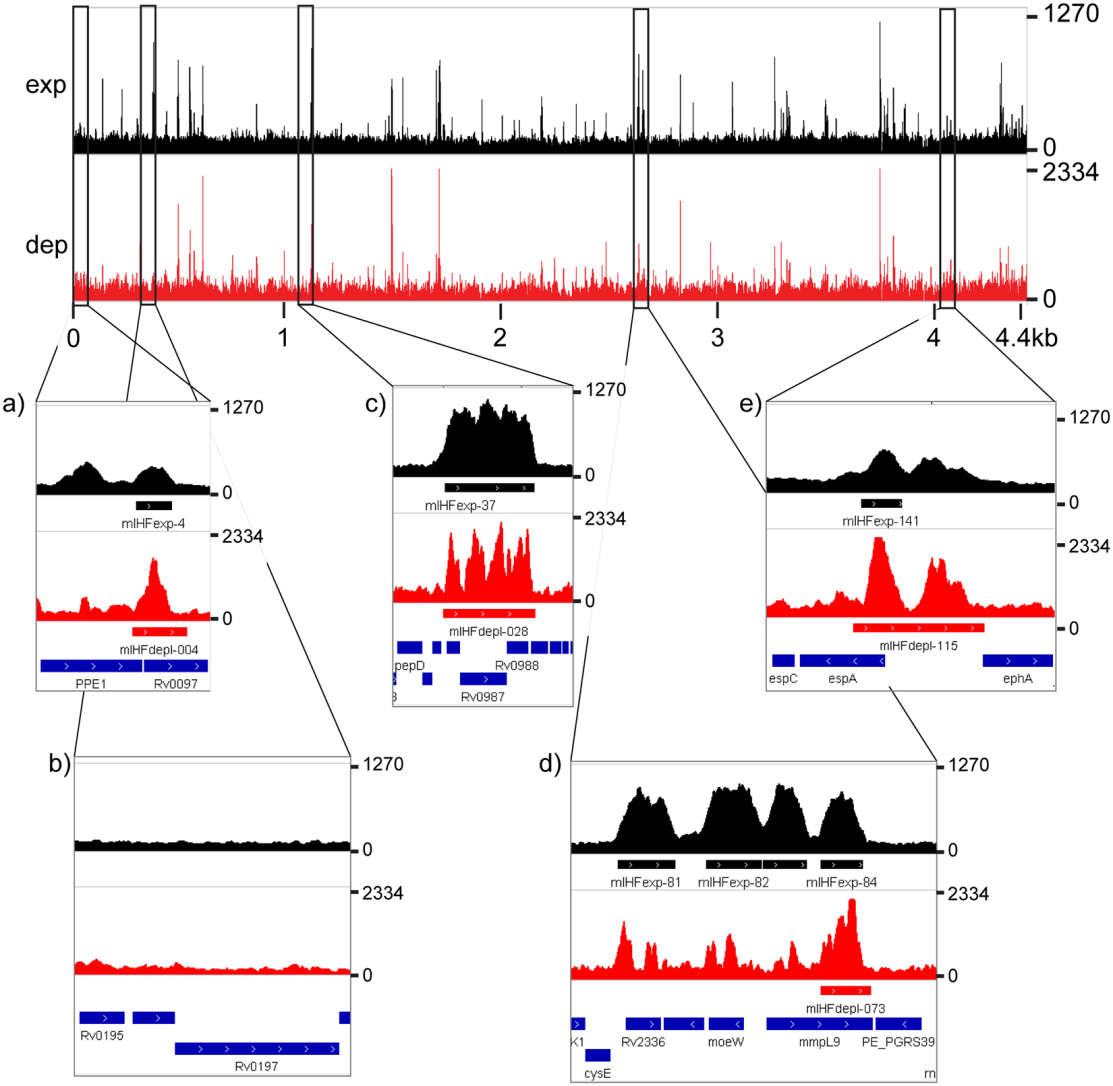
mIHF contacted regions on the H37Rv chromosome. The upper panel shows the global distribution of mIHF in exponentially growing cells (exp, in black) and in mIHF-depleted cells (dep, red). The number of reads is indicated on the y-axis, while gene length and names are shown on the x-axis. Zoomed images show examples of mIHF binding. (**a**) mIHF peak at *rv0097*, whose transcription is 10.5-fold downregulated upon mIHF depletion. (**b**) No peak is located close to the top upregulated genes *rv0196* and *rv0197*. (**c**) Third top-scoring peak cluster close to *mprA*/*mprB*. (**d**) Accumulation of peaks around *mmpL9*. (**e**) *EspA* extended promoter region.

Loci enriched for mIHF were distributed all over the genome, with some regions harbouring several peaks next to each other. Figure 10 shows the binding profile for mIHF in exponential growth phase and after depletion, with regions of interest highlighted. The genomic region around the top upregulated genes *rv0196*-*rv0197* was not bound by mIHF (Fig. 10b), while the long intergenic region upstream of the most downregulated gene *espA* showed peaks in both conditions (Fig. 10e).

Despite being named integration host factor, mIHF did not bind close to either of the prophages PhiRv1 (spanning *rv1573*-*rv1587*) or PhiRv2 (*rv2650*-*rv2659*) in the chromosome. On the other hand, the so-called genomic islands (GI) defined by Becq *et al*.^22^, which represent 4.5% of the whole *M. tuberculosis* genome, were contacted by mIHF in both growth conditions. Almost one quarter (23%) of mIHF binding sites in exponential phase, and 18% after depletion, were near GI.

The impact of mIHF as a potential direct activator or repressor was defined for features where mIHF peaks either overlapped the genes or were within 500 bp of the coding sequence boundaries. Genes associated with an mIHF binding site were more frequently deregulated as compared to genes unlinked to an mIHF-enriched locus (p < 0.0001). Genes associated with mIHF following depletion had a mean expression level of −1.91, representing an almost 2-fold downregulation, compared to genes unaffected by mIHF depletion with a mean expression level of 0.05, indicating no transcriptional change. Similarly, genes in close proximity to mIHF binding sites in exponential phase were expressed at −2.0, and more distant genes at 0.02. This indicates that mIHF binding directly impacts gene regulation.

## Discussion

NAPs have a major effect on bacterial gene expression by acting as global transcription regulators and chromosome architects. In *M. tuberculosis*, three NAPs - EspR, Lsr2 and HupB - were characterized in depth previously, while the function of a fourth NAP candidate, Rv3852, was disproved^17^. In this report, the systematic investigation of mIHF is presented and its potential importance for *M. tuberculosis* pathogenesis described. Like EspR^7^, mIHF is exclusively cytosolic, and present throughout the bacterial growth cycle, which contrasts with the findings of Sharadamma *et al*., who found increased mIHF levels in stationary phase^23^. The number of mIHF binding sites (153) was similar to that of EspR (165), but less than the 305 loci contacted by Lsr2^24^. No common motif was found for the mIHF binding sites, confirming the non-specific DNA-binding demonstrated *in vitro*^12^ and the broad mIHF ChIP-peaks may indicate binding to structured DNA rather than to a specific sequence.

The ATc-dependent silencing of *mihF* expression confirmed the predicted essentiality of the gene and demonstrated that mIHF is required not only for multiplication but also for bacterial survival. Indeed, a dramatic effect on cell morphology and global physiology was noticed upon depletion of the protein. At least two passages were necessary to decrease mIHF levels and this is consistent with the remarkably high abundance of mIHF reported in exponential phase as well as during hypoxia-induced dormancy^25^. Intriguingly, despite the effects in cell elongation, cell shape and septum formation observed upon mIHF depletion, no gene known to be involved in these processes was heavily deregulated. Expression of *ftsZ*, coding for the cell division initiator protein, and *wag31*, whose product localizes to the septum^26^, were marginally downregulated, whereas *ftsK* and *ftsQ*, necessary for septum formation^27^, were only slightly upregulated.

Overall, genes harbouring an mIHF binding site within their coding sequence or located less than 500 bp away were downregulated upon mIHF depletion although mIHF peaks were still detected. The mIHF binding profiles in exponentially growing cells as well as in non-permissive conditions were essentially the same but differed in peak width since after mIHF depletion the peaks were generally narrower. The strong, but not complete, correlation between mIHF peaks in exponential phase (153) and after depletion (124), with 62 overlapping signals, suggests that mIHF probably binds to a core set of genes with higher affinity. Examples are represented by the *rv0097* and *espA* loci, which were found to be more enriched in the depleted condition than in the exponentially growing phase. The numerous differentially regulated genes identified upon mIHF depletion indicated a pleiotropic effect with a strong bias towards virulence-related genes. Most prominent was the 20-fold repression of one of the main virulence operons, *espACD*, which is critical for ESX-1 function, EsxA/B secretion and full virulence of *M. tuberculosis*^28^.

The region preceding the *espACD* operon, where mIHF binds, has been deleted from the genome of some tubercle bacilli during evolution of the *M. tuberculosis* complex and is referred to as region of difference 8, RD8^29^. It is especially striking that RD8 contains binding sites for numerous regulatory proteins including the NAPs EspR, Lsr2 and mIHF, as well as the activator CRP. In addition, RD8 is contacted by the response regulators MprA^30^ and PhoP of two different two component systems, MprAB and PhoPR^31^. To overcome mutations in PhoR, that decrease virulence, L6 strains of *M. africanum* and all animal-adapted tubercle bacilli have deleted RD8 thereby removing a regulatory layer and restoring full virulence^31^.

In the same vein, expression of PE11 (*rv1169c*, LipX), which is necessary for survival of *M. tuberculosis* in *ex vivo* models^32^, was repressed 18-fold following mIHF depletion. LipX modulates glycolipid-synthesis^33^, its downregulation reduces PDIM (phthiocerol dimycocerosate) levels in *M. tuberculosis*, leading to enhanced cellular aggregation and biofilm formation^34^. PDIMs are required for phagosomal escape^35^ and to do so they act concertedly with ESX-1^36^. Thus, another important virulence factor is controlled by mIHF. Biofilms are associated with persistent infections of *M. tuberculosis*^37^ and contain mycolic acids in their extracellular matrix^38^. In addition to *lipX*, several genes encoding mycolic acid tailoring enzymes (e.g. *umaA*, *mmaA3*) were expressed at lower levels in mIHF-depleted cells, implying that mIHF also affects biofilm formation and mycolic acid synthesis, consistent with the results of our GO survey. Furthermore, expression of *ppe17*, situated 17 bp downstream of *lipX*, was repressed 13.5-fold. PPE17 was shown to be upregulated in macrophages^39,40^, again indicating the importance of mIHF for successful infection.

Apart from control of virulence genes, RNA, DNA and protein synthesis pathways were also found to be deregulated. For example, the gene for the epsilon subunit (*dnaQ*) of DNA polymerase III was 3.2-fold upregulated, while *dnaN* and *dnaA* were 4.6- and 5.7-fold repressed, respectively. DnaN acts as a bridge between the alpha and epsilon subunit and plays a regulatory role on the replicase^41^. Absence of the DnaA chromosomal replication initiator and DnaN proteins should reduce initiation of DNA replication, and this is consistent with the lower nucleic acid synthesis observed by uracil-incorporation.

Most of the sigma factors were not differentially expressed upon mIHF depletion, except for *sigE*, which was >4-fold downregulated. This alternative sigma factor is activated in various stress conditions, such as inside macrophages^42^, and is required for interrupting phagosome maturation^43^. SigE positively regulates *mprAB* and induces the stringent response in *M. smegmatis*^44^. As a consequence of the downregulation of *sigE* expression, neither the stringent response nor the SOS response to DNA damage were induced upon mIHF depletion, which implies that mIHF does not play a direct role in facing starvation. Concerning protein synthesis, the low amount of tRNAs and downregulation of ribosomal protein genes were reflected in the poor incorporation of radiolabelled leucine by mIHF-depleted cells.

Taken together, our results show that in *M. tuberculosis* mIHF is an essential, highly stable, cytosolic NAP whose gene repertoire overlaps with that of several other NAPs and regulatory proteins. mIHF likely influences gene expression by organizing chromatin structure and this will be probed using single cell analysis and chromatin conformation capture techniques to determine whether it acts alone or in association with other chromatin regulators.

## Methods

### Strains, media and chemicals

*M. tuberculosis* H37Rv and *mihF*-cKD strains were grown at 37 °C either in Middlebrook 7H9 broth (Difco) supplemented with 10% albumin-dextrose-catalase, 0.2% glycerol and 0.05% Tween 80 or in Sauton’s liquid medium supplemented with 0.005% Tween 80. Cultures were plated on Middlebrook 7H10 (Difco) agar supplemented with 10% oleic acid-albumin-dextrose-catalase and 0.2% glycerol. Hygromycin (50 μg ml^−1^), kanamycin (25 µg ml^−1^), streptomycin (25 μg ml^−1^), 2.5% sucrose or Anhydrotetracycline (ATc, Clontech, 600 ng ml^−1^) were added when needed. For cloning procedures, One shot® TOP10 chemically competent *Escherichia coli* (Invitrogen) were grown in Luria–Bertani (LB) broth or on LB agar with hygromycin (200 μg ml^−1^), kanamycin (50 μg ml^−1^) or spectinomycin (25 μg ml^−1^). All chemicals were purchased from Sigma-Aldrich, unless otherwise stated. Experiments involving *M. tuberculosis* have been carried out in a Biosafety Level 3 (BSL3) laboratory, according to the national and international guidelines (Authorization number A070027/3).

### Plasmid and conditional knockdown mutant construction

In order to promote homologous recombination, 1 kbp up- and downstream regions of full length *mihF* were generated by PCR amplification using primers *mihF*-UF/*mihF*-UR and *mihF*-DF/*mihF*-DR (listed in Supplementary Table 1) respectively. Fragments were ligated in-frame with the AvrII site and cloned into the PacI and AscI sites of pJG1100, resulting in the suicide vector pCS35. The complementing plasmid pCS31 was constructed by cloning the full-length *mihF* gene, amplified with primers *mihF*-F/*mihF*-R, into pGA44 under control of the repressible *ptr* promoter^45^. The two genes located downstream of *mihF* (*gmk* and *rpoZ*), were amplified with primers *gmk*-F/*rpoZ*-R. The constitutively active promoter P*furA*102^46^ was amplified with primers P*furA*102-F/P*furA*102-R. An overlap PCR was performed to fuse P*furA*102 with *gmk-rpoZ*, and then cloned into pCS31, resulting in pNO12. The complementing vectors harbouring *mihF*-80 and *mihF*-86, as well as P*furA*102-*gmk*-*rpoZ*, were constructed in pGA118, a derivative of pGA44, which carries a hygromycin instead of a streptomycin resistance cassette, resulting in pNO62 and pNO63, respectively. Expression of *gmk* and *rpoZ* by the complementing vector was necessary to compensate for the polar effects caused by the deletion of the *mihF* gene.

Deletion of the full-length *mihF* gene was obtained by homologous recombination using plasmid pCS35. After transformation of *M. tuberculosis* H37Rv, the first recombination event was selected on 7H10 plates, supplemented with hygromycin and kanamycin. Colonies were screened by colony PCR using CS-402/CS-403 and CS-404/CS-405 primer pairs. The merodiploid strain was generated by integration of plasmid pNO12 at the L5 *attB* site and transformants were plated on 7H10 with hygromycin, kanamycin and streptomycin. Finally, deletion of the wild type gene by allelic exchange and generation of the *mihF* cKD strain was accomplished by plating the bacteria on 7H10 supplemented with streptomycin and 2.5% sucrose. The resulting colonies were tested by PCR with primers CS-403/CS-415 for deletion of *mihF* from its native locus and confirmed by Southern blot.

### Genomic DNA extraction and Southern Blot

Mycobacterial genomic DNA was extracted using standard protocols. To confirm successful allelic exchange of *mihF*, genomic DNA was digested with AvrII and PvuII restriction enzymes. DNA fragments were separated by 0.8% agarose gel electrophoresis before capillary blotting onto a Hybond-N+ nylon membrane (GE Healthcare) and hybridization with a probe corresponding to the same upstream and downstream regions of *mihF* cloned into pJG1100. Hybridization was carried out using the ECL Direct Nucleic Acid Labelling and Detection System (GE Healthcare) as recommended by the manufacturer.

### Growth curve measurements, colony forming unit counts, 3H-leucine and 3H-uracil incorporation

To characterize the growth of the *mihF* cKD mutant, the strains were grown to mid-logarithmic phase and then diluted to an optical density at 600 nm (OD_600_) of 0.05 in 7H9 medium. ATc was added to 600 ng ml^−1^ and the OD_600_ was recorded at different time points to obtain the growth curves. As ATc is light sensitive and depleted over time, cultures were diluted to OD_600_ = 0.05 every three days.

Nucleic acid and protein synthesis were measured by incorporating tritium-labelled leucine and uracil as previously described^47^. As mycobacteria do not incorporate exogenous thymidine, but can use uracil for RNA as well as for DNA synthesis after methylation^47^, only uracil was used to assess total nucleic acid production. Strains H37Rv with ATc, *mihF*-cKD with and without ATc (600 ng ml^−1^) were grown to mid-exponential phase, diluted to OD_600_ = 0.1 and then subsequently diluted again to the same OD = 0.1 every day. Of these cultures, 1 ml was incubated daily with 1 µCi ^3^H-uracil and ^3^H-leucine, respectively. After 24 h, the sample was washed once in PBS supplemented with 0.05% Tween 80, the cells were harvested by centrifugation and stored at −80 °C until further processing. Counts per min were measured by suspending the sample in 5 ml Ecoscint XR (National diagnostics) on a Beckman Coulter LS6500 Multi-Purpose Scintillation Counter. At every sampling point, the number of colony-forming units (CFU) per millilitre of culture was evaluated and protein samples were taken at days 0, 2, 5 and 9. Cumulative protein and nucleic acid incorporation was derived as the sum of the daily incorporation multiplied by the CFU for the total incorporation relative to day 0, multiplied by the dilution factor, to account for the growth rate during the 24 h incubation. CFU were evaluated from serial dilutions of *M. tuberculosis* cultures plated on 7H10 plates and cumulative CFU calculated similarly by summing the previous CFU with the daily CFU multiplied by the dilution factor necessary to reach OD = 0.1 to normalize all three samples. Growth rate was calculated as the daily OD_600_ divided by the target OD_600_ of 0.1.

### Scanning electron microscopy

For surface scanning electron microscopy, *mihF-*cKD mutant without ATc and H37Rv wild type strains were grown in 7H9 until mid-exponential phase, pelleted, washed in PBS and resuspended to OD_600_ = 0.5. The *mihF*-cKD mutant with ATc was diluted three times in fresh ATc-containing medium and then subjected to the same protocol. Samples were then fixed on a coverslip in a solution of 1.25% glutaraldehyde, 1% tannic acid in phosphate buffer (0.1 M, pH = 7.4) for 1 h, washed in PBS prior to fixing for 30 min in 1% osmium tetroxide. The samples were then dehydrated in a graded alcohol series and dried by passing through the supercritical point of carbon dioxide (Leica Microsystems CPD300) and coated with a 2 nm layer of osmium metal using an osmium plasma coater (Filgen OPC60). Scanning electron microscopy images were taken using a field emission scanning electron microscope (Merlin, Zeiss NTS) with an acceleration voltage of 2 kV and the in-lens secondary electron detector. Cell length of bacteria was measured in ImageJ (n = 110 for H37Rv wild type, n = 96 for *mihF*-cKD without ATc and n = 98 for *mihF*-cKD + ATc). Two-tailed, one-way analysis of variance (ANOVA) with Kruskal-Wallis post-test was performed with 303 degrees of freedom and F value = 184.2.

### Fluorescence microscopy

Strains H37Rv and *mihF*-cKD were grown to exponential phase in 7H9 Middlebrook media without ATc, while *mihF*-cKD plus ATc was diluted three times in fresh medium with 600 ng ml^−1^ ATc. Samples were incubated with SYTO9 (4 µM), which stains the DNA, and FM4–64 (5 µg ml^−1^) to stain the membrane for 20 min at 37 °C. Bacteria were mounted on an agarose pad and imaged with an Olympus IX81 microscope under a 100x objective. Representative images were selected and single channel and composite images were adjusted for brightness and contrast in ImageJ.

### Total RNA extraction and 5′ rapid amplification of cDNA ends

*M. tuberculosis* H37Rv and *mihF*-cKD cultures were harvested by centrifugation, pellets were resuspended in TRIzol Reagent (ThermoFisher) and stored at −80 °C until further processing. Total RNA was extracted by bead-beating as previously described^48^. Integrity of RNA was checked by agarose gel electrophoresis, purity and amount of RNA were assessed using a Nanodrop instrument and Qubit Fluorometric Quantitation (ThermoFisher) respectively. SuperScript III First-Strand Synthesis System (Invitrogen) was used to generate randomly primed cDNA from 500 ng of RNA, according to the manufacturer’s recommendations. Primers CS-057/CS-058 for *sigA* were used to normalize the amount of cDNA template added to each sample.

For the 5′-RACE, 2 µg of *M. tuberculosis* H37Rv RNA and 1 µg of primer NO-095 were incubated at 70 °C for 5 min and then at 55 °C for 1 h in the presence of 1x cDNA synthesis buffer, 1 mM dNTPs, 40 U RNase inhibitor, 25 U Transcriptor Reverse Transcriptase (5′/3′ RACE Kit, 2nd Generation, Roche). cDNA was then purified with the High Pure PCR Product Purification kit (Roche), and used in the subsequent poly(A) tailing reaction (30 min at 37 °C in the presence of 0.2 mM dATP and 80 U Terminal Transferase, Roche). Semi-nested PCR amplification on poly(A)-tailed cDNA was performed using an oligo dT-anchor primer (CS-080) and primer NO-094. Three amplification products were obtained, cloned into pTOPO (Invitrogen) and sequenced.

### RNA sequencing and analysis

RNA was extracted from biological duplicate samples from exponential phase H37Rv and three-times diluted *mihF*-cKD as described above. The ribosomal RNA was depleted with the Ribo-Zero rRNA Removal Kit for Gram-positive Bacteria (Illumina), following the manufacturer’s instructions. Libraries were prepared by the Lausanne Genomic Technologies Facility, using the Truseq Stranded mRNA Library Prep kit reagents (Illumina) according to the manufacturer’s recommendations. The multiplexed libraries were sequenced on a Hiseq. 2500 instrument using TruSeq SBS Kit V4 reagents as single-end 100 nt-long reads. Sequencing data were processed using the Illumina Pipeline Software version 1.84. Reads were adapter- and quality-trimmed with Trimmomatic v0.33^49^. The quality settings were “SLIDINGWINDOW:5:15 MINLEN:40”. Reads were aligned with Bowtie2^50^. Counting reads over annotated features was done with featureCounts^51^. Annotation was taken from TubercuList release R27 (http://tuberculist.epfl.ch/). Differential gene expression analysis was done using DESeq2^52^.

For gene ontology (GO) enrichment analysis, a cut-off of 4-fold differentially expressed genes was chosen. The GO annotation was retrieved from BioCyc^53^ and analysis was performed with TopGO^54^. The conservative weighted algorithm and Fisher’s exact test were used to calculate p-values for enrichment of GO terms in biological processes, molecular functions and cellular components in up- and downregulated genes upon mIHF depletion. The tree was pruned to a node size of 5 to exclude statistical artefacts of small sized GO terms.

### Protein extraction, immunoblot analysis and subcellular fractionation

*M. tuberculosis* cells grown in 7H9 were pelleted at different time points by centrifugation, washed once in Tris-Buffered Saline (TBS, 20 mM Tris-HCl pH 7.5, 150 mM NaCl) and stored at −80 °C until further processing. Cells were sonicated in TBS supplemented with a protease inhibitor tablet (cOmplete, mini, EDTA free, Roche) for 15 min and the protein solution was then sterilized by filtration through a 0.2 µm filter to remove any residual intact cells. Protein samples were quantified using the Qubit Fluorometric Quantitation device (ThermoFisher). Equal amounts of protein preparations were loaded on SDS-PAGE 12–15% NuPAGE gels (Invitrogen) and transferred onto PVDF membranes using a semidry electrophoresis transfer apparatus (Invitrogen). Membranes were incubated in TBS-Tween blocking buffer (25 mM Tris pH 7.5, 150 mM NaCl, 0.05% Tween 20) with 5% w/v skimmed milk powder for 3 h at 4 °C prior to overnight incubation with primary antibody. Membranes were washed in TBS-Tween three times, and then incubated with secondary antibody for 2 h before washing. Signals were detected using Chemiluminescent Peroxidase Substrate 1 (Sigma-Aldrich).

Primary monoclonal anti-mIHF antibody was produced by Alere against recombinant mIHF-86 and used at a concentration of 1:2,000 in immunoblots. Horseradish peroxidase (HRP) conjugated Goat anti-mouse Kappa (SouthernBiotech) secondary antibody was used at a 1:12,000 dilution. Anti-RpoB antibodies (NeoClone) were used to detect RpoB, the internal loading control. Band intensity of immunoblots was analysed with Fiji/ImageJ and normalized to the intensity of the RpoB signal.

Cell fractions were obtained as described previously^55^. Briefly, H37Rv was grown in Sauton’s medium with 0.005% Tween 80 to mid-exponential phase, cells were collected by centrifugation, and supernatant was filtered and concentrated 100 x to obtain the secreted fraction. The pellet was treated with 0.25% Genapol-X080 for 30 min followed by centrifugation at 14,000 g for 10 min and the proteins of the resulting supernatant precipitated with TCA, yielding the capsular fraction. The remaining pellet was subjected to sonication to break the cells, sterilized by filtration through a 0.2 µm filter followed by ultra-centrifugation at 45,000 rpm for 1 h. The supernatant contained the cytosolic fraction, while the pellet was enriched with membrane proteins.

### Chromatin Immuno Precipitation (ChIP) and library construction

ChIP was performed as previously described^56^. Briefly, exponentially growing *M. tuberculosis* H37Rv liquid cultures (for input control, EspR ChIP and mIHF ChIP) or mIHF depleted *mihF*-cKD mutant were cross-linked with formalin 1% for 10 min and quenched with glycine 125 mM for 10 min, washed twice in Tris-buffered saline (TBS, pH 7.5) and sonicated on a Diagenode Bioruptor with 30 sec on/off cycles for 10 min on high settings to shear DNA to 200–500 bp fragments. Immunoprecipitation was performed with monoclonal anti-mIHF (Alere) or polyclonal rat anti-EspR antibodies (Statens Serum Institut, Copenhagen, Denmark) in 1 ml immunoprecipitation (IP) buffer (containing 50 mM HEPES-KOH pH 7.5, 150 mM NaCl, 1 mM ethylendiaminetetraacetic acid (EDTA), 1% Triton X-100, 0.1% (w/v) sodium deoxycholate, 0.1% sodium dodecyl sulphate (SDS) and one protease inhibitor cocktail tablet (Roche)) overnight at 4 °C. 100 µl Dynabeads sheep anti-rag IgG (Dynal Biotech) for EspR and 100 µl per sample anti-ProteinL magnetic beads (Pierce) for mIHF were pre-saturated with 1 mg ml^−1^ bovine serum albumin and 0.1 mg ml^−1^ salmon sperm DNA, then incubated with each corresponding sample for 4 h. The IP was washed 5 times with increasing stringency buffers as described previously^7^. Input control was not incubated with antibody. Libraries were prepared with the NEBNext Ultra II DNA kit (NEB) by the Lausanne Genomic Technologies Facility following the manufacturer’s instructions, multiplexed and sequenced on a Hiseq 2500 instrument.

### ChIP-seq data analysis

Alignment was performed with Bowtie2^50^ against the H37Rv genome (NCBI NC_000962.2) and resulted in 8.1 M uniquely aligned reads for EspR, 1.1 M for mIHF in exponentially growing cells, 7.3 M for mIHF in mIHF depleted cells and 3.8 M for the input, respectively. For mIHF ChIP in exponentially growing cells, two different concentrations of antibodies were tested. These two datasets were pooled, as the Spearman correlation of 0.96 was excellent (Fig. S5). HOMER^57^ was used for peak calling using the dynamic peak size algorithm with the input as a control. The enrichment is calculated relative to the input sequence, which might not reflect the real peak size. Resulting peaks were manually curated, subsequently annotated and further analysed with BEDtools^58^ and deepTools^59^. Profile plots of mIHF binding sites in exponentially growing cells and in mIHF-depleted cells, as well as GC content of H37Rv were generated with deepTools at binsize = 25 and visualized with IGV^60^. Coordinates of transcriptional start sites were taken from Cortes *et al*.^18^. CRP binding sites were extracted from^61^ and Lsr2 from^24^. Minch *et al*. identified 305 Lsr2 binding sites by ChIP-seq, the same technique used here for mIHF and EspR. Additionally, the Minch *et al*. binding sites represent a subset of the >800 sites identified by an earlier ChIP-chip experiment^62^, we decided to use the Minch *et al*. dataset for our analysis. ChIPPeakAnno was used to find overlapping peaks. Connected peaks were merged, resulting in an overall lower sum of peaks for each transcription factor^63^.

Genes associated with mIHF binding sites (<500 bp distance to feature boundary) were analysed by ANOVA with Bonferroni’s multiple comparison test to test for a correlation in gene expression. N = 331 for genes associated with mIHF binding and n = 3785 for genes not close to a mIHF peak in mIHF depleted cells. N = 330 for genes associated with mIHF binding in exponential phase, and n = 3831 for genes without any mIHF peak in close proximity. F-value was 147.2 and degrees of freedom = 8276. GC content of binding sites was extracted from the H37Rv genome (NC_000962.3), with n = 162, 153 and 124 for EspR binding sites, mIHF binding sites in exponentially growing cells and mIHF binding sites in mIHF-depleted cells, respectively. All 4111 features were analysed for the overall GC content of *M. tuberculosis*. One-way, two-tailed ANOVA with Bonferroni’s multiple comparison test was performed in GraphPad Prism with an F-value of 583.7 and 4549 degrees of freedom.

## Electronic supplementary material

Supplementary Information

Supplementary Dataset 1

## Data Availability

Raw and processed data of RNA- and ChIP-seq data are available at the NCBI Gene Expression Omnibus repository under accession number GSE111194, https://www.ncbi.nlm.nih.gov/geo/query/acc.cgi?acc = GSE111194.
