# Supplementary material for: Essential Nucleoid Associated Protein mIHF (Rv1388) Controls Virulence and Housekeeping Genes in Mycobacterium tuberculosis
Source: Sci Rep. 2018 Sep 21;8:14214. doi: 10.1038/s41598-018-32340-2 (PMC6155035; doi:10.1038/s41598-018-32340-2)
Supplement: Supplementary file 1 — Supplementary Information [file 41598_2018_32340_MOESM1_ESM.pdf]

Essential Nucleoid Associated Protein mIHF (Rv1388) Controls Virulence and Housekeeping Genes in *Mycobacterium tuberculosis*

Nina T. Odermatt, Claudia Sala, Andrej Benjak, Stewart T. Cole

École Polytechnique Fédérale de Lausanne, Global Health Institute, Station 19, 1015 Lausanne, Switzerland

Text S1

The actual length of the *mihF* gene has been under debate since Mishra et al proposed an alternative transcriptional start site (Mishra 2013). In this study, we identified the bona fide coding sequence of *mihF*, which starts 255 bp downstream of the annotated translation start site. Upstream of transcription start site (TSS) 3, we detected a -10 promoter element as well as a ribosomal binding site (Fig. S1). Furthermore, our complementation studies showed that the full-length protein could not be downregulated by addition of ATc, suggesting the presence of alternative promoters (TSS1 and/or TSS2 in Fig. S1) in the cloned fragment, which compensated for *ptr* silencing. These findings are supported by previously published RNA-seq data (Uplekar, 2013), where expression of *mihF* starts downstream of the annotated translational start site. Further evidence for the smaller size of mIHF comes from several mass spectrometry experiments conducted by various groups (Målen, 2010; de Souza, 2011; Schubert, 2013), which only detected peptides belonging to mIHF-86 and not to the N-terminal-extended version. The discrepancy between our findings and those of Sharadamma et al. might therefore be attributed to a wrong assumption of the molecular mass of mIHF. Indeed, upon overexposure, an immunoblot showed a non-specific band at ca. 22 kDa that is recognised by the anti-mIHF antibodies. These bands might have been misinterpreted as mIHF (Fig. S6). In a subsequent study, the same group discovered that only the C-terminal part of mIHF, that means the part corresponding to mIHF-86, was able to bind DNA, but still claimed that the extra 86 amino acids are part of the mIHF protein (Sharadamma, 2017). Recently, a new gene essentiality analysis in *M. tuberculosis* proposed that *mihF* is not essential, but transposons were only inserted into the N-terminal part of the annotated *mihF* (Dejesus, 2017), which is not included in the genuine coding sequence. These data additionally confirm that *mihF* translation starts downstream of its annotated start site. Taken together, we can confidently propose the alternative translational start site of *mihF* at 1,563,949 on the H37Rv chromosome, 255 bp downstream of the annotated start of the *mihF* gene.

Fig S1

TSS1 - 91 bp

AACCATGGCGCGACACGCGCGATAGGTCGCCCAACCGCGAGTGATCCCCGGCACTGCGAGTTGCGACGCCACCTGCCGCCACCAGTCGT

+1 *mihF*

CGGCCGTCGTCGACCGGTTGAGCAGGTCCGGAAAGCCGAAATCCA**T**TGTTAGGCAACACTATTCATGTCCCATGCCAGCCATGCCGGCA

TSS2 + 127 bp

CGGACACGGGGCTCCGTCGAGAGGCCTTCGAGGTCGCCCGGCGGACCGCTGGCCGGTGGCACGTGCTACTCCCACGCTGCACGTTTGTC

TANNT -10 TSS3 + 167 bp

CCCAAAACCAGGGGGTCGGGTTAGATTTCGTCAGGAAGCCTGAGTACGGTCGTCTGCGCTGGCCGGCGTACCCGGCCGGGACAAACAAC

+1 *mihF*-80 SD +1 *mihF*-86

GATCGATTGATATCG**A**TGAGAGACGGAGGAATC**G**TGGCCCTTCCCCAGTTGACCGACGAGCAGCGCGCGGCCGCGTTGGAGAAGGCTGC

TGCCGCACGTCGAGCGCGAGCAGAGCTCAAGGATCGGCTCAAGCGTGGCGGCACCAACCTCACCCAGGTCCTCAAGGACGCGGAGAGCG

ATGAAGTCTTGGGCAAAATGAAGGTGTCTGCGCTGCTTGAGGCCTTGCCAAAGGTGGGCAAGGTCAAGGCGCAGGAGATCATGACCGAG

CTGGAAATTGCGCCCACCCGCCGCCTTCGTGGCCTCGGTGACCGTCAGCGCAAGGCCCTGCTGGAAAAGTTCGGCTCCGCCTAA

Fig. S1. *mihF* gene locus. Annotated translation start site (+1 *mihF* corresponds to the first base of the full length *mihF*), and the two proposed short forms of *mihF* (*mihF*-80 and *mihF*-86). The three identified transcription start sites (TSS) by 5’-RACE are marked by arrows and the potential AG-rich Shine-Dalgarno (SD) ribosomal binding site and TANNT -10 motifs are highlighted in grey.

Fig S2

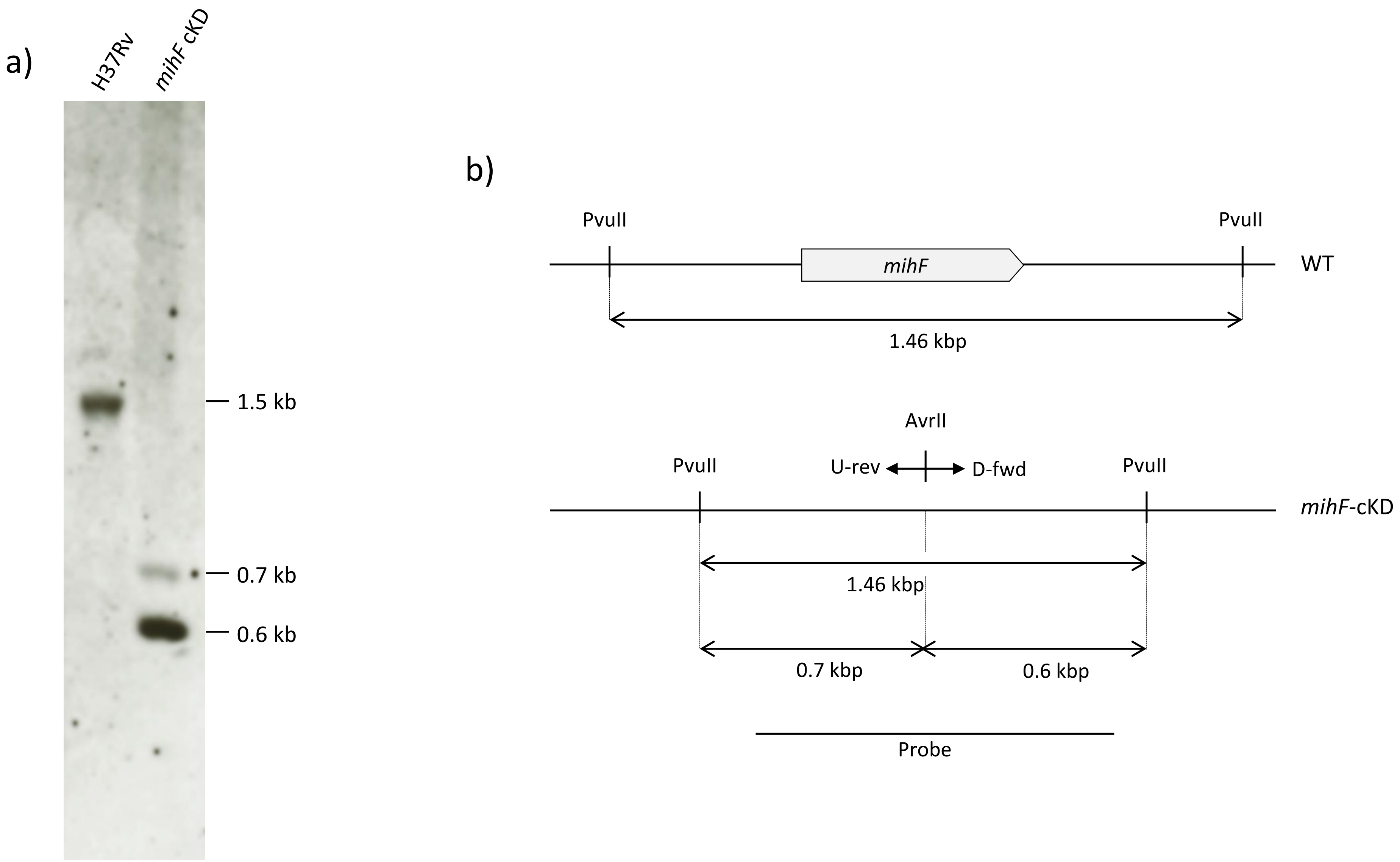

Fig. S2. *mihF-cKD* mutant construction. a) Southern blot analysis of *mihF*-cKD and H37Rv parental strains and genomic locus with restriction sites and probe used for Southern blot (b).

Fig. S3

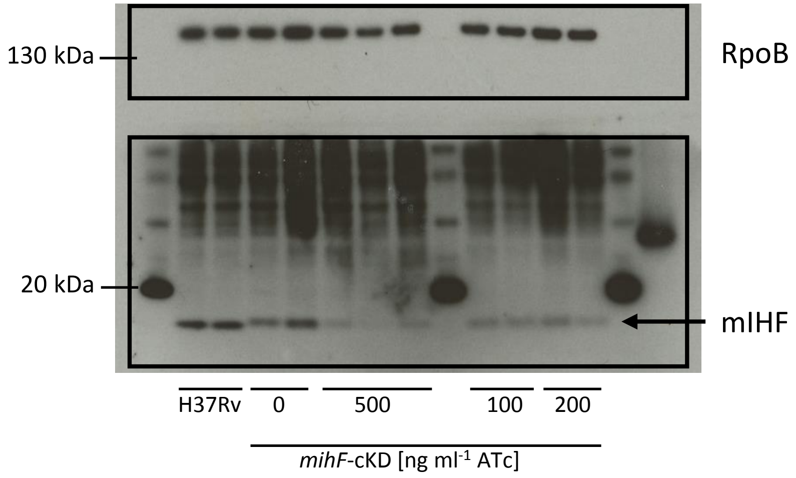

Fig. S3. mIHF levels upon *mihF* silencing by ATc. Immunoblot of mIHF upon *mihF* silencing by ATc. Duplicate or triplicate (in case of *mihF*-cKD with 500 ng ml^{-1} ATc) samples were taken after 2 dilutions (9 days).

Fig. S4

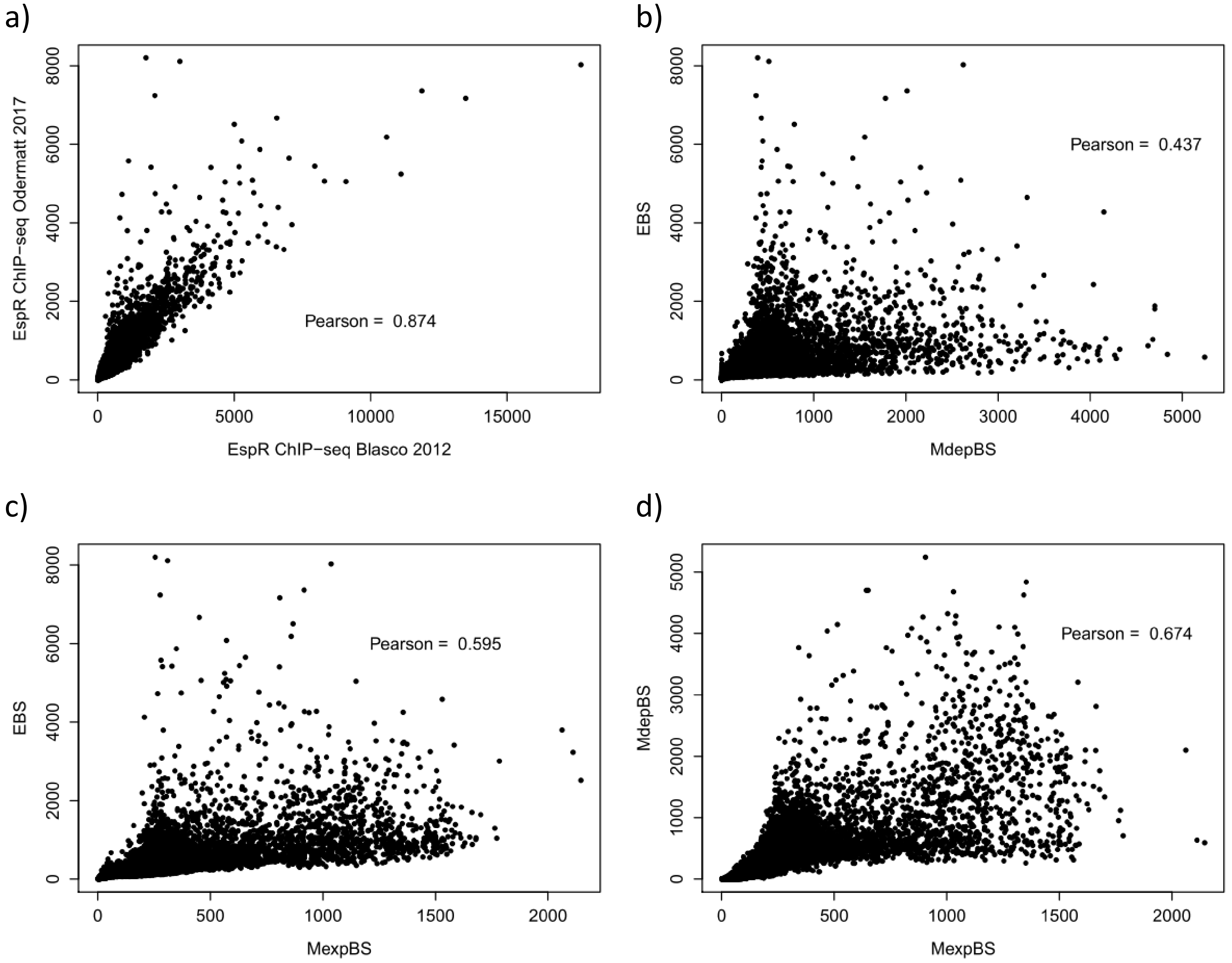

Fig. S4. Correlation between different ChIP-seq experiments. a) Correlation between the 2012 EspR ChIP-Seq by Blasco et al. and the EspR ChIP-seq conducted in this study has a Pearson index of 0.87. b) and c) Correlation between mIHF binding sites in mIHF depleted condition (MdepBS) versus EspR binding sites (Pearson index = 0.44) and between exponentially growing cells (MexpBS) and EspR (Pearson index = 0.6). d) Correlation between MexpBS and MdepBS with a Pearson index = 0.67.

Fig. S5

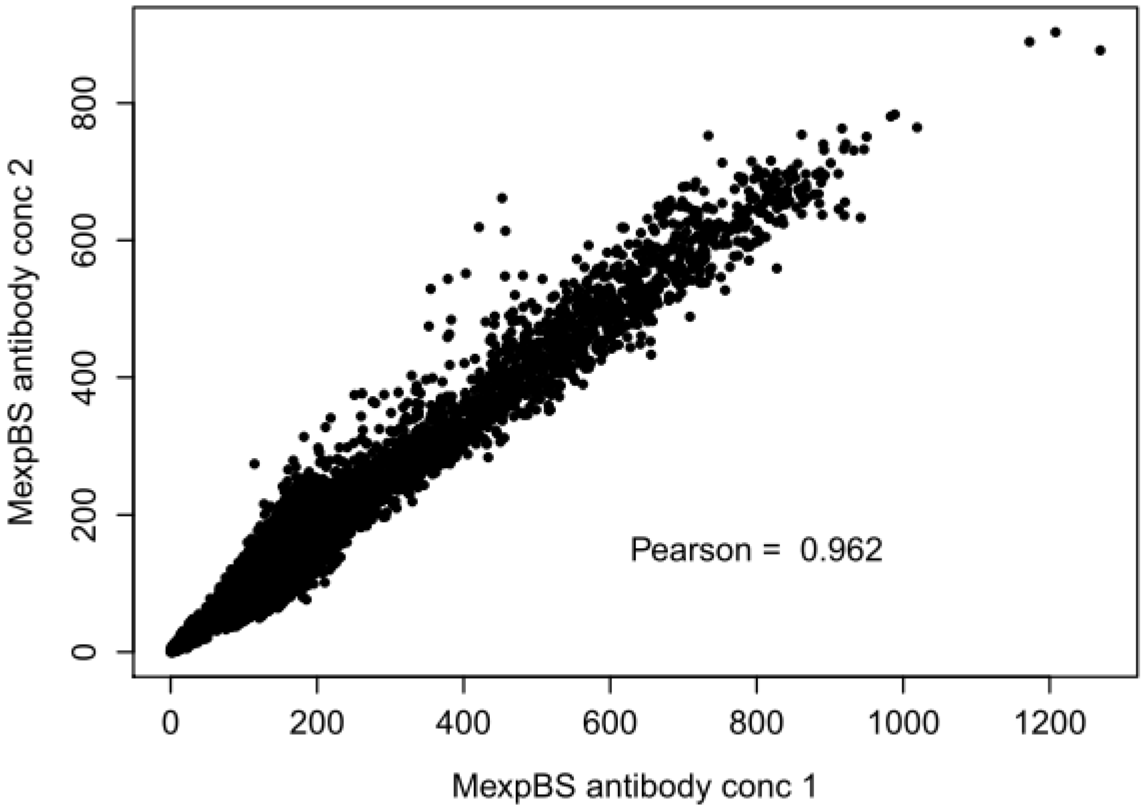

Fig. S5. Correlation between ChIP-seq with two different amount antibodies. mIHF binding sites in exponentially growing cells (MexpBS) in two independent experiments were conducted with different concentrations of primary anti-mIHF antibody. Data were pooled because of the excellent correlation (Pearson index = 0.96).

Fig. S6

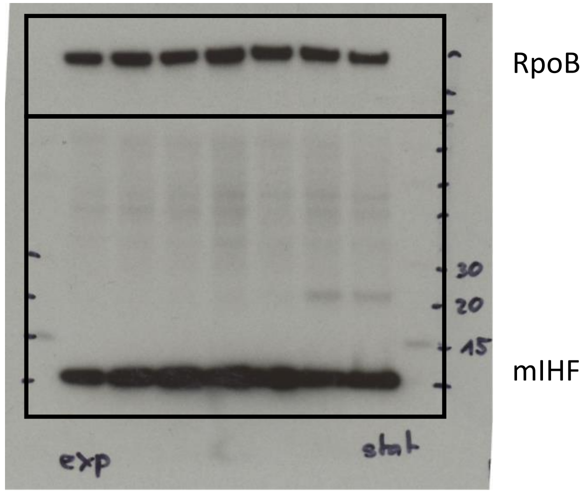

Fig. S6. Immunoblot of mIHF. An overexposed immunoblot with anti-mIHF antibodies reveals a weak band at ca. 22 kDa appearing at stationary phase. Samples were taken at day 2 (OD_{600} = 0.45), 4 (1.16), 7 (1.41), 10 (clumpy), 16, 22 and 32.

Fig. S7

b)

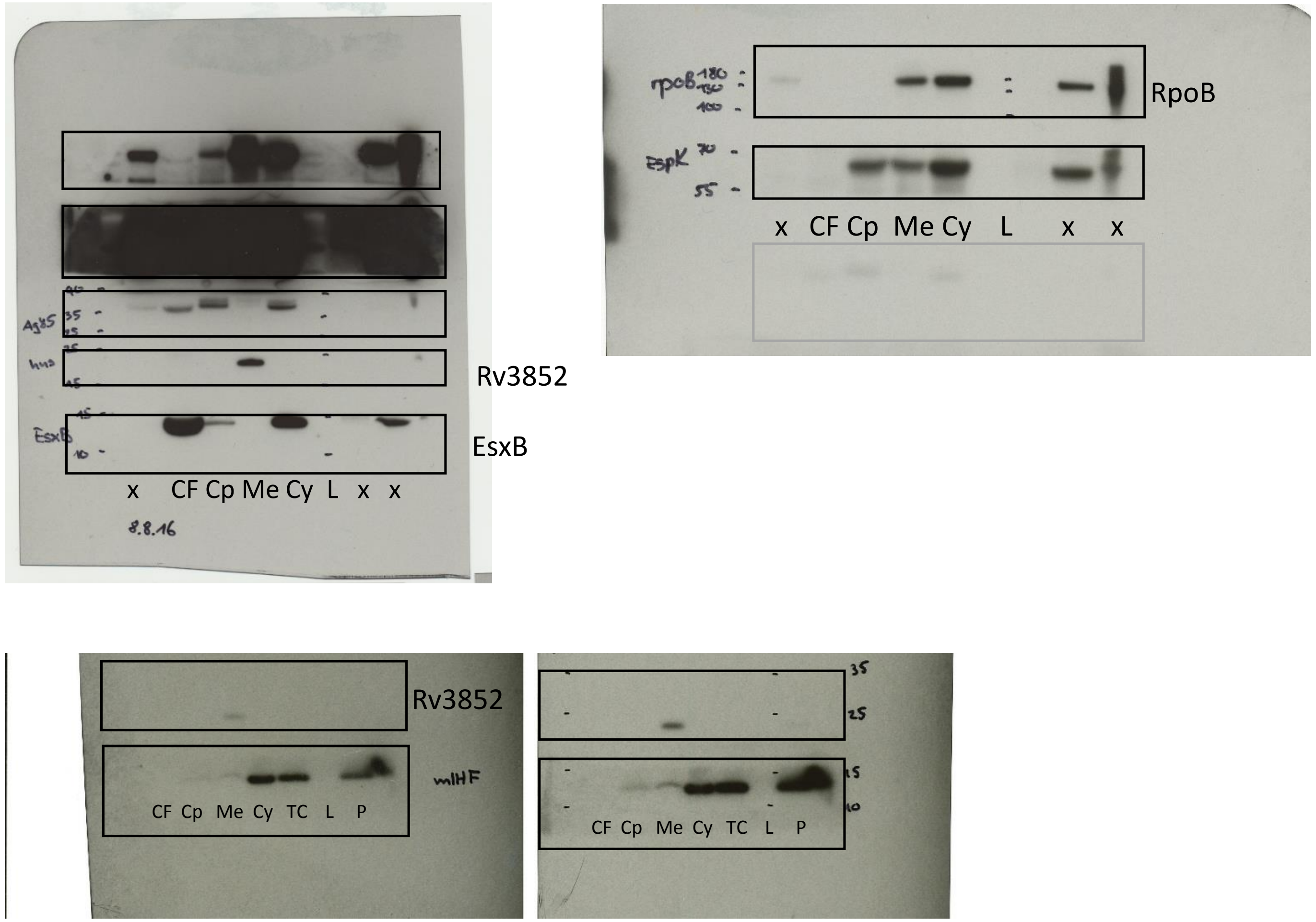

a)

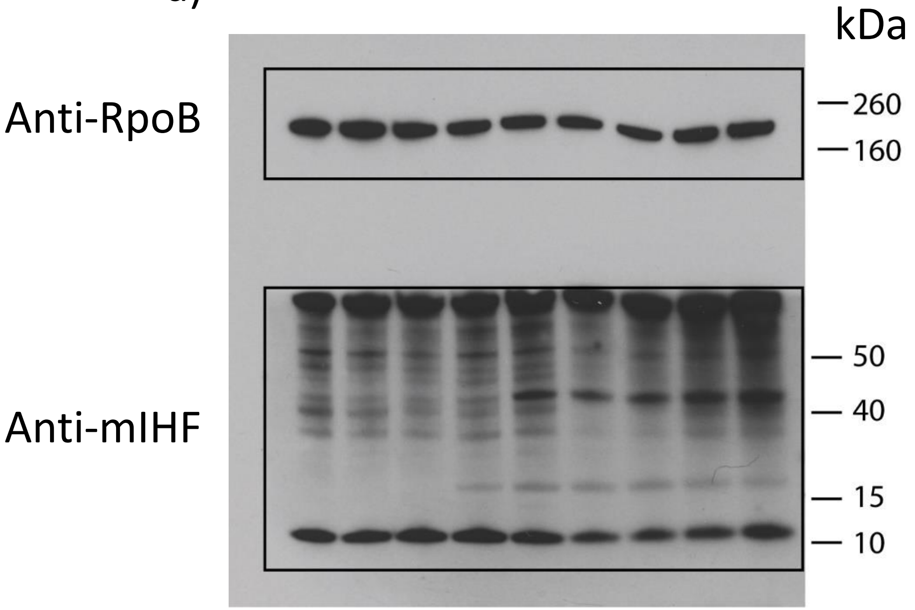

Fig. S7. Full immunoblots. Non-cut immunoblot with anti-RpoB and anti-mIHF antibodies of Figure 1a) is shown in S7a). The black rectangles depict the border of the cut membrane. S7b) Shows the full images of the cut membranes of the fractionation blots in Fig 1b), top leftpanel: EsxB and Rv3852, top right panel: new scan of RpoB blot. Culture filtrate (CF), capsule (Cp), membrane (Me) and cytosolic fraction (Cy) are indicated. x represent test fractions of other experiments, L indicates the ladder. Bottom panel: mIHF blot with different exposure times. Culture filtrate (CF), capsule (Cp), membrane (Me), cytosolic fraction (Cy), total cell extract (TC), Ladder (L) and purified mIHF protein (P) are indicated.

Supplementary Table 1. Primers used in this study.

Primer name	Sequence (5’ → 3’)
**CS-080-dTanchor**	GAC CAC GCG TAT CGA TGT CGA CTT TTT TTT TTT TTT TTV
**CS-364-mihFUF**	ACG TTC TTA ATT AAC CCG CAG TTG CTG TCG GCT GCC C
**CS-365-mihFUR**	ACG TTC CCT AGG CAA TGG ATT TCG GCT TTC CGG ACC TGC
**CS-366-mihFDF**	ACG TTC CCT AGG TAA CCC CGC CGG CCG ACG ATG
**CS-367-mihFDR**	ACG TTC GGC GCG CCC CCT CGC CAA GCT GGT TGT AG
**CS-370-mihFF**	ACG TGT CCT AGG TTA GGC AAC ACT ATT CAT GTC CCA TGC
**CS-371-mihFR**	ACG TGT GGC GCG CCT TAG GCG GAG CCG AAC TTT TCC AGC
**CS-402-U1**	CAG ATC GCT ACC TGG CTG GGC
**CS-403-D1**	GGT ACG CCC ACC ACA GGC
**CS-404-U2**	CAC CAG TCG TCG GCC GTC GTC
**CS-405-D2**	GCG GAC CGA CAT ATT CGA GGA TG
**CS-415-mihFF**	GAC GCA ACC ATG GCG CGA C
**CS-423-PfurA102F**	ACG TGA TTC GAA CCA CCA TGC AGG CCC GGC
**CS-424-PfurA102R**	GGT CCC TCG CCG ACG CTC ACA CTA GAC AAT ATG ACT CCC
**CS-425-gmkF**	GGG AGT CAT ATT GTC TAG TGT GAG CGT CGG CGA GGG ACC
**CS-426-rpoZR**	ACG TCC ATG GCT ACT CGC CCT CGG TGT GCT CG
**mihF-80-F**	CGA TCC TAG GAT GAG AGA CGG AGG AAT CGT GG
**mihF-86-F**	GCA TCC TAG GGT GGC CCT TCC CCA GTT GAC CG
**NO-094-rev-120**	GCG TCC TTG AGG ACC TGG G
**NO-095-rev-180**	GGC AAG GCC TCA AGC AGC G

Supplementary Table 2. Plasmids used in this study.

Plasmid name	Description	Reference
**pJG1100**	Suicide vector for mutant construction, HygR, KanR, sacB	Gomez & Bishai, 2000; Munoz-Elias et al., 2006
**pGA44**	Integrative vector at L5 attB site, carrying the TET-PIP OFF expression system, StrR	Kolly et al., 2014
**pGA80**	pMV261-derived vector, carrying the L5 int gene for expression in trans, lacking oriM, KanR.	Kolly et al., 2014
**pGA118**	Integrative vector at L5 attB site, carrying the TET-PIP OFF expression system, HygR	Kolly, unpubl.
**pCS35**	Suicide vector for mutant construction derived from pJG1100, HygR, KanR, sacB	This study
**pCS31**	Full-length mihF cloned in pGA44, integrative vector at L5 attB site, StrR	This study
**pNO71**	Empty vector pGA44 carrying PfurA102-gmk-rpoZ	This study
**pNO12**	Full-length mihF, PfurA102-gmk-rpoZ cloned in pGA44, integrative vector at L5 attB site, StrR	This study
**pNO62**	mihF-80, PfurA102-gmk-rpoZ cloned in pGA118, integrative vector at L5 attB site, HygR	This study
**pNO63**	mihF-86, PfurA102-gmk-rpoZ cloned in pGA118, integrative vector at L5 attB site, HygR	This study
